# Modern MRI Diagnostics of Upper-Extremity-Related Nerve Injuries—A Prospective Multi-Center Study Protocol for Diagnostics and Follow Up of Peripheral Nerve Injuries

**DOI:** 10.3390/jpm12101548

**Published:** 2022-09-20

**Authors:** Martin Aman, Daniel Schwarz, Annette Stolle, Konstantin Davide Bergmeister, Arne H. Boecker, Simeon Daeschler, Martin Bendszus, Ulrich Kneser, Leila Harhaus

**Affiliations:** 1 Department of Hand-, Plastic and Reconstructive Surgery, Burn Center, BG Trauma Center Ludwigshafen, Department of Hand- and Plastic Surgery, University of Heidelberg, Ludwig-Guttman Str. 13, 67071 Ludwigshafen, Germany; 2 Department of Neuroradiology, Neurological University Clinic, Heidelberg University Hospital, Im Neuenheimer Feld 400, 69120 Heidelberg, Germany; 3 Karl Landsteiner University of Health Sciences, Dr. Karl-Dorrek-Straße 30, 3500 Krems, Austria; 4 Department of Plastic, Aesthetic and Reconstructive Surgery, University Hospital St. Poelten, 3100 St. Poelten, Austria; 5 Center for Restoration of Extremity Function, Department of Surgery, Medical University of Vienna, Waehringer Guertel 18-20, 1090 Vienna, Austria

**Keywords:** plastic surgery, reconstructive surgery, microsurgery, nerve damage, MRI, neurosonography

## Abstract

(1) Background: Peripheral nerve injuries are severe injuries with potentially devastating impairment of extremity function. Correct and early diagnosis as well as regular regeneration observation is of utmost importance for individualized reconstruction and the best possible results. Currently, diagnoses and follow-up examinations are based on clinical examinations supported with electroneurography, which often causes delays in treatment and can result in impaired healing. However, there is currently no diagnostic device that can reliably correlate the anatomic–pathological parameters with the functional–pathological changes initially and during therapy. With new technologies such as MR neurography (MRN), precise visualization of potential nerve damage and visualization of the reinnervation processes is assumed to accelerate clinical decision making and accompaniment of individualized treatment. (2) Methods/Design: This prospective clinical study will examine 60 patients after peripheral nerve lesion aged 18–65 years from trauma timepoint onward. Patients should be observed over a period of 18–24 months with regular clinical examinations, electroneurography, and ultrasound to compare the potential of MRN to current gold-standard diagnostic tools. Furthermore, 20 patients with the same inclusion criteria stated above, with an internal fixation and osteosyntheses of humerus fractures, will be examined to determine the visibility of peripheral nerve structures in close proximity to metal. (3) Discussion: Peripheral nerve injuries are often accompanied with severe, expensive, and long-lasting impairment of extremity function. An early and precise diagnosis of the nerve lesion, as well as the healing course, is crucial to indicate the right therapy as soon as possible to save valuable time for nerve regeneration. Here, new technologies such as MRN aim to visualize nerve injuries on fascicular level, providing not only early diagnosis and therapy decisions, but also providing a precise tool for monitoring of reinnervation processes. As severe injuries of a nerve are often accompanied with bone fractures and internal fixation, we also aim to evaluate the visualization feasibility of nerves in close proximity to metal, and ultimately improve the outcome and extremity function of patients after a peripheral nerve injury.

## 1. Background

Traumatic nerve lesions of the upper extremities occur especially in young adults. In approximately 15% of all cases, nerve lesions occur as result of an occupational accident [1,2,3]. The trunk nerves (median, ulnar, or radial nerve) of the upper extremity are hereby predominantly affected [1,4,5,6].

Nerve lesions are primarily diagnosed clinically which can be supplemented by technical examinations such as electrophysiology or neurosonography [7]. Each of these instruments provides individual parameters about the nerve damage and its regeneration. The nerve conduction velocity evaluates the propagation of nerve impulses and neurosonography visualizes the structure of the nerves. Due to this indirect assessment, a correct determination is often difficult due to ambiguous findings and complex anatomy (superficial vs. deep structures), or the simultaneous occurrence of several combined pathologies (e.g., mixed nerve lesion stage VI according to Sunderland, modified by Mackinnon [8]). An early and precise diagnosis of the lesion is essential for the right choice of therapy strategy and is thus decisive for the success of the treatment in peripheral nerve injuries. Improved diagnostics using MR neurography could help to make therapy decisions more precise, thus reducing the duration of sick leave (average 26.8 days) and high therapy costs of more than 300,000 affected Europeans per year [1,2,3].

However, there is currently no widely established diagnostic device that can reliably correlate the anatomic–pathological parameters (e.g., partial or full lesion of the nerve) with the functional–pathological changes (muscle denervation, neuroma formation) initially and during the course of therapy. Therefore, to this day, the structured clinical examination of the patient is still the most essential part for the diagnosis and follow-up of nerve lesions. The Hoffmann–Tinel sign in particular provides information about the proceeding of the newly sprouting axons and thus about the location of the reinnervation level [9].

In everyday clinical practice, it is an established procedure to have a follow-up appointment about every 3 months to clinically assess the reinnervation. As a result, valuable time is lost in a large number of cases until a potentially necessary nerve reconstruction is performed. Potential consecutive permanent functional losses with medical (restricted function) and financial (inability to work, unemployment) consequences can result.

So far, this problem could not be solved using conventional MRI examinations, since the limited spatial resolution of several millimeters is not sufficient to adequately visualize nerves. For this reason, conventional MRI has so far played a subordinate role in the routine diagnosis of nerve lesions and has only been used for visualizing damage to large nerves (e.g., sciatic nerve) or the nerve plexus (brachial plexus), yet it is still unable to provide any information about the function of a nerve.

Modern MR neurography represents a further development in conventional MR examinations with higher resolution and innovative analysis software. It can thus display the anatomical and the functional parameters of nerves, providing a potential diagnostic tool of high value for the future.

MRN is promising due to a high spatial resolution due to special magnetic coils, and combined with latest software advances, it provides better insights in the visualization of nerves.

This prospective multi-center study examines the clinical application of MR neurography for the optimized primary and follow-up diagnosis of peripheral nerve lesions of the upper extremities.

## 2. Aim of the Study

The aim of this study is the prospective evaluation of MR neurography for the diagnosis and follow up of the reinnervation process of:

1. Peripheral nerve lesions of the trunk nerves of the upper extremity (60 patients);
2. Applicability and nerve visualization in patients with internal plate osteosynthesis of humeral fractures (20 patients).

We aim to determine specificity and sensitivity of MR neurography in correlation to the standard examinations (gold standard: clinical and intraoperative findings, electroneurography, and sonography) of acute–traumatic nerve injuries. Hereby, a baseline of MRN is created and correlated to the most reliable diagnostic tool, the intraoperative findings. With electroneurography and clinical examination, the course of regeneration can be assessed and correlated to the MRN.

The patient data collection for this takes place at the respective study centers (center A and center B) and the analysis of specificity and sensitivity, as well as the diagnostic algorithm at center A. MR neurography is performed at center C.

## 3. Methods

This prospective clinical study will examine a total of 60 male and female patients aged 18–65 years after peripheral nerve lesions over a period of 48 months. Patients should be observed from the initial presentation for a period of 18 months in the case of nerve lesions distal to the elbow, and 24 months in the case of nerve lesions proximal to the elbow. The additional time of the examination results from the duration of the nerve regeneration (1 mm per day) and the longer regeneration distance.

Furthermore, 20 patients with an internal fixation and osteosynthesis after humeral fracture and no nerve lesion with the same inclusion criteria stated above will be examined to determine the visibility of peripheral nerve structures in close proximity to metal.

The study is approved by the local ethics committee, and we ask potential participants for written consent prior of inclusion to the study.

## 4. Participants

During the study period, patients with the following nerve pathologies should be examined in addition to standard diagnostics using MR neurography:

Acute traumatic nerve lesions of the upper extremity;

Trunk nerves from the exit from the brachial plexus to the distal carpal tunnel;

Radial nerve;

Median nerve;

Ulnar nerve;

Musculocutaneous nerve.

The target group of this study comprises patients with injuries to the trunk nerves of the upper extremity (60 patients) as well as 20 patients with inserted osteosynthesis material without nerve lesion (illustration of nerve structure close to osteosynthesis material).

Inclusion criteria:General:
Male or female older than 18 and younger than 65 years;Communication in German or English possible;Signed declaration of consent.
Nerve pathologies:
Fresh (<96 h) open–traumatic nerve injuries to the trunk nerves of the upper extremity: radial, ulnar, median, musculocutaneous nerves, and their branches from the brachial plexus to the distal end of the carpal tunnel.
Osteosynthesis:
Patients with an MRT-compatible plate fixation using a tita-nium plate of the upper extremity;Humeral fracture;No nerve lesion necessary.
Exclusion criteria
Rejection of study participation;Age <18 or >65 years;∙Failure to show up for a follow-up examination;Patients who are unable to provide information or who are unconscious;Simultaneous participation in another study to evaluate a drug or medical device;Vitally threatening injury upon initial diagnosis (e.g., multiple trauma);Insufficient knowledge of German or English;Mental health issues, which limits patients’ capacity to consent (e.g., acute psychosis, dementia);Pregnancy, breastfeeding;Ongoing immunosuppressive or antineoplastic therapy.
Absolute contraindications to MRI
Pacemaker;Mechanical heart valves;Brain and spinal cord stimulators as well as most other electrical stimulating devices implanted in the body;Insulin pumps or other drug pumps;Ventriculoperitoneal shunts (VP shunts);Cochlear implants;Foreign metal bodies in the soft tissues of the body, e.g., in the eyes, in the abdominal or chest cavity;Obesity, which prevents the use of the MRI;Upper-limb immobility that prevents an MRI scan.
In the case of a relative contraindication, only after the patient has been informed and the radiologist performing the procedure has given their consent
Claustrophobia;Prosthetic joint replacement;Tattoos;Piercings;Vascular stents, e.g., in the coronary arteries.


## 5. Interventions

Acute–traumatic nerve lesions are usually open injuries that are treated directly using epineural sutures.

After the initial diagnosis of an acute–traumatic nerve lesion and treatment in accordance with guidelines, a doctor in the respective clinic provides comprehensive information about the study. If the patient agrees to be included in the study, they will be included and given their written consent in accordance with the inclusion and exclusion criteria.

After the patient has been included, the supervisor/head of the study is notified, who then takes on the further planning. Participation in the study does not replace or delay existing therapies. The treatment is identical to that of patients outside the study. After nerve lesion, MR neurography is carried out within 72 h to further supplement the diagnosis. An MRI contrast agent is used to improve visualization.

After the patients have been included in the study, conventional nerve diagnostic examinations are carried out in patients with nerve lesions (clinical examination by the responsible center as well as neurography and neurosonography by a consultant neurologist affiliated with the center with proven expertise and advanced training in the field of peripheral nerves, especially neurography and neurosonography) at the respective study center including additional MR neurography examinations at center C at the 4–5 times mentioned below.

For the second group, patients with fractures of the humerus are treated by osteosynthesis. After internal fixation, patients are evaluated for inclusion or exclusion and assessed with the MRN. In patients after osteosyntheses MRN is carried out a minimum of 1 month after operation.

MR neurography has the same low risk profile as conventional MRI examinations. Due to the technical requirements (MRI coils) and the necessary user knowledge, this MRI examination is currently only offered in few centers.

## 6. Objective

This prospective clinical study examines the diagnostic specificity and sensitivity of MR neurography in correlation to the previous standard examinations (gold standard: clinical and intraoperative findings, neurography, and possibly sonography) of acute–traumatic nerve injuries. Furthermore, the course of nerve regeneration is assessed and correlated to clinical and electrophysiological findings to create a radiological “baseline” of the visualization of nerve regeneration in MRN. In addition, its applicability for nerve diagnosis after osteosynthesis is evaluated.

## 7. Primary Research Question

Is MRN able to precisely visualize the correct diagnosis of a peripheral nerve injury and clinical progression of reinnervation?

## 8. Secondary Research Question

Is MRN able to precisely visualize the nerve despite the presence of osteosynthesis?

## 9. Hypotheses

Alternative hypothesis (H1):

MR neurography shows a high sensitivity (>85%) to detect nerve lesions correctly.

MR neurography objectifies nerve lesions earlier (within 72 h) than electrophysiology (only positive from about 1 week) and neurosonography (especially with deep lesions).

MR neurography is able to visualize nerve structures in close proximity to metal.

Null hypothesis (H0):

MRN is not able to detect nerve lesions correctly and cannot detect lesions earlier than electrophysiology.

MR neurography is not able to visualize nerve structures in close proximity to metal.

## 10. Design

This study uses a prospective longitudinal design including patients at two centers in Germany. Due to ethical reasons, no randomization is performed.

## 11. Outcomes—Investigations

Patients with attached osteosynthesis material.

**Figure 1:**
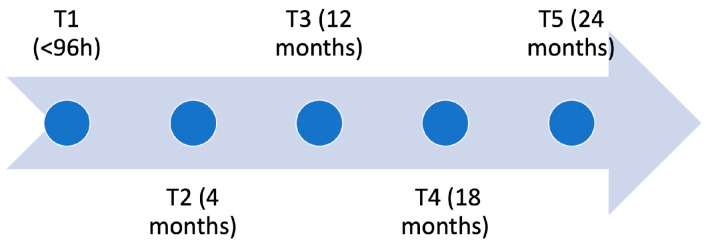
Timeline of the trial. At all timepoints, patients are tested with methods described in Table 2.

MR neurography is carried out once with the special interference-suppressing analyses, 2–12 weeks after implantation. Since these patients do not suffer from nerve lesions, no additional clinical examination or questionnaires are applied. (Table 1; Figure 1)

## 12. Statistical Considerations and Sample Size

The number of study cases for the study objectives was calculated on the basis of the scientific literature for the planning of diagnostic pilot studies [10,11,12]. Therefore, the number of cases is calculated as follows: Sensitivity of MR neurography was assumed to be 85% based on internal examinations. We therefore calculate the sample size with a 95% confidence interval using the method described by Eng. J [11].

Sample size calculation with: (4 × (1.96)^2^ × 0.85 × 0.15)/(0.2^2^) = 48.98 patients. In order not to risk a loss of power with an expected dropout of approximately 10%, we add a safety margin of 10%. This results in 60 patients for the main study group (acute–traumatic nerve lesions).

Criteria for discontinuing are met if the patient drops out voluntarily or does not attend follow-up examinations.

As there is no data for the visualization of nerves in proximity to metal, no sample size calculation could be performed for the second part. This part has the character of a pilot study.

## 13. Randomization

Due to ethical reasons, no randomization is planned. The assignment to individual centers depends on regional emergency care strategies.

## 14. Statistical Methods

The MR neurography diagnosis of nerves can be represented as a numerical value between 0 and 1 using fractional anisotropy (FA). The first small pilot studies on healthy people showed that a high FA value correlates with intact nerve function. The Pearson correlation coefficient will be used to correlate the FA with the intraoperative findings (% of the severance).

A *t*-test is used to determine the dependence of the FA on the clinical and neurosonographic binary variables (presence of sensory deficit, presence of motor deficit, presence of a defect in neurosonography), provided the data are normally distributed. Otherwise, corresponding nonparametric tests are used. In order to examine the variables over time, the following evaluation methods are used: ANOVA for the dependence of the FA change on the clinical–binary parameters, and partial correlation is used to estimate the relationship between changes in FA and changes in nerve conduction velocity. Due to the hypothesis-generating study design of this pilot study, no correction for multiple testing is applied [13]. A *p*-value of 0.05 or less is established as statistical significance in accordance with good scientific practice for all investigations. The study results are evaluated with SPSS V21 software from IBM (for further information also see Supplementary Materials-spirit checklist).

## 15. Timeframe—Recruiting Plan

A total of 4 years is planned for the realization of this study.

## 16. Discussion

Peripheral nerve injuries are devastating injuries, often resulting in severe impairment of extremity function. So far, some factors relevant to optimal nerve regeneration have been identified [14,15,16]. Hereby, correct and early diagnosis of the nerve damage is of utmost importance in order to enable early-stage therapy.

New developments in more powerful MRI scanners and new evaluation algorithms, such as diffusion tensor imaging (DTI), have recently shown groundbreaking advances in the diagnosis and localization of nerve damage [17]. The term MR neurography [18] summarizes techniques that, in comparison to clinical and electrophysiological examinations, were able to localize nerve lesions more precisely in smaller studies. DTI can, in many cases, visualize the nerve lesion exactly and show reinnervation signs and muscle de- and reinnervation [19].

For this purpose, peripheral nerves are evaluated non-invasively by means of the phenomenon of FA (diffusion co-effects). The visualization of this physical effect is used in order to measure the directional preference of the water proton propagation. This can be used to indicate nerve pathologies and to see the spread of masses as an indirect indication of intact nerve pathways. This technique is already used successfully in CNS relationships such as Alzheimer’s disease or multiple sclerosis [20,21].

We further compare the MRN to standard clinical examinations to obtain a better understanding of the progression of nerve regeneration seen in MRI studies and the corresponding clinical presentation. Currently, there is no “baseline” of knowledge about radiological representation of functional nerve regeneration in MRN. With this prospective approach, we aim to provide this “baseline” by correlating it to the most valuable diagnostic tool, the intraoperative findings. With that knowledge and the standard of care follow-up examinations such as electroneurography and the clinical examination, a better understanding of radiological representation of DTI and FA should be provided.

For a comprehensive understanding of the psychological burden of the patients suffering from peripheral nerve injuries and their individual regeneration process, we assess multiple factors such as pain, depression, and the functioning of upper extremities in daily life activities. Current clinical-standard assessments such as electrophysiological testing are conducted not only to obtain an idea of possible correlation to MRI findings but also as a security precaution to ensure the best possible treatment for participants in case of inferior results being seen in the MRN.

The best treatment for patients suffering is strongly dependent on a multidisciplinary and early diagnosis and treatment. With new technologies such as the MRN, we will accelerate diagnosis and visualize regeneration after treatment. This will improve the outcome due to earlier intervention in cases of non-regeneration.

## 17. Trial Status

Recruitment started in January 2019 and is expected to be completed in January 2023.

## Figures and Tables

**Table 1:** Assessment of patients with nerve lesions.

Standardized Clinical Examination	
Clinical report form for anamnesis	Standardized questionnaires for medical anamnesis and follow-up examinations
Clinical report form for sensory motor functioning testing	The results of the following tests are recorded: Strength grades according to the classification of the British Medical Research Council from 0 = paralysis to 5 = normal strength; Hand strength using a Jamar dynamometer: grip force and the 3-point grip are evaluated; Preliminary sensory testing of both forearms and hands in a side-by-side comparison; Tactile detection threshold using the Weinstein Enhanced Sensory Test (WEST), the tactile detection threshold is ascertained at index areas for each trunk nerve at the hand. Hereby, the monofilaments (200 g, 4 g, 2 g, 0.2 g, 0.07 g) are placed on the skin in descending order and a defined pressure is applied; Two-point discrimination is recorded using a standardized Dellon discriminator in descending order. The distance between the pins varies from 1 mm to 8 mm. The static threshold is tested by placing the pins vertically on the skin and the dynamic threshold by pulling the pins over the skin; Localization of the Hoffmann–Tinel sign by lightly tapping; Range of motion.
**Technical Examinations**	
Electrophysiology Neurosonography MR neurography	Measurement is performed by trained neurologists affiliated to the trauma center and comprises the following procedures: Motor neurography (muscle sum action potential, distal motor latency, nerve conduct velocity); Sensory neurography (sensitive nerve action potential, nerve conduct velocity); Somatosensory evoked potentials using needle electrodes; Electromyography (quantification of pathological spontaneous activity and arbitrary activity). Measurement by trained neurologists affiliated with the trauma center. Measurement performed by a trained radiologist at center C.
**Standardized Questionnaires**	
Short Form 36 (SF-36) Morfeld et al., 2011	The SF-36 is a well-established self-reported measure of health-related quality of life. In addition to the general state of health, the content of the SF-36 is the occurrence of pain and the impairment in everyday life due to mental health problems. The values can vary between 0 and 100 points; lower values reflect poorer well-being, higher values reflect better well-being.
Impact of Event Scale—Revised (IES-R) Horowitz et al., 1979	The IES-R is a 22-item self-disclosure questionnaire that assesses the subjective distress caused by traumatic events. Respondents are asked to identify a specific stressful life event and then indicate how much they were worried or disturbed by it in the last 7 days. The items are rated on a scale from 0 (“not”) to 4 (“extreme”). The IES contains three subscales: intrusion, avoidance, and hyperarousal.
Disabilities of Arm, Shoulder, and Hand Scale (DASH) Germann et al., 2003	The German version of the DASH is regarded as the standard for the subjective assessment of limitations in the functionality of the upper extremities. The questionnaire consists of 30 items, scoring from 1 to 5. Optionally, there is a sports and music module (4 items) and a work and occupation module (4 items). Using an algorithm, the raw data are transformed into a DASH score between 0 (no restrictions) and 100 (maximum restrictions).
PainDETECT Freynhagen et al., 2006	The PainDETECT tool was developed by the German Research Association for Neuropathic Pain. It allows screening for the presence of neuropathic pain. It comprises 9 questions and records the intensity, pattern, and quality of pain. Its sensitivity and specificity are over 80 percent.
Depression–Anxiety–Stress Scale (DASS21-G) Nilges and Essau, 2015	The DASS is a self-disclosure questionnaire that assesses depression, anxiety, and stress without confounding somatic factors. The short version DASS 21G consists of three scales, depression, anxiety, and stress, each with 7 items scoring from “0—did not apply to me at all” to “3—applied very much to me or most of the time”.

**Table 2:** Times of clinical and technical examinations for patients with nerve lesions.

	T1 within 96 h after Injury	T2 4 Months after Injury	T3 12 Months after Injury	T4 18 Months after Injury	T5 24 Months after Injury (only Injuries Proximal to the Elbow)
CRF anamnesis	X	X	X	X	X
Sensory and motor function testing	only healthy site	X	X	X	X
**Technical examination**					
Electrophysiology		X	X	X	X
MR neurography	X	X	X	X	X
Neurosonography	X	X	X	X	X
**Questionnaires**					
SF-36	X	X	X	X	X
IES-R	X	X	X	X	X
DASH	X	X	X	X	X
PainDETECT	X	X	X	X	X
DASS	X	X	X	X	X

## Data Availability

Not applicable.

## Supplementary Materials

The following are available online at https://www.mdpi.com/article/10.3390/jpm12101548/s1, Spirit Checklist.

## List of Abbreviations

DASH	Disability of the Arm, Shoulder and Hand
DASS	Depression, Anxiety and Stress Score
DRF	clinical report form
DTI	diffusion tensor imaging
FA	fractional anisotropy
IES-R	Impact of Event Scale—short
MRI	magnetic resonance imaging
MRN	magnetic resonance neurography
SF-36	Short Form 36
