# Supplementary material for: Modern MRI Diagnostics of Upper-Extremity-Related Nerve Injuries—A Prospective Multi-Center Study Protocol for Diagnostics and Follow Up of Peripheral Nerve Injuries
Source: J Pers Med. 2022 Sep 20;12(10):1548. doi: 10.3390/jpm12101548 (PMC9604736; doi:10.3390/jpm12101548)
Supplement: Supplementary file 1 [file jpm-12-01548-s001.zip › jpm-1802998-supplementary.pdf]

SPIRIT

STANDARD PROTOCOL ITEMS: RECOMMENDATIONS FOR INTERVENTIONAL TRIALS

SPIRIT 2013 Checklist: Recommended items to address in a clinical trial protocol and related documents*

Section/item	Item No	Description
**Administrative information**		
Title	1	Modern MRI diagnostics of upper extremity related nerve injuries- a prospective multi-center study protocol for diagnostics and follow up of peripheral nerve injuries
Trial registration (page 13 of the manuscript)	2a	Deutsches Register Klinischer Studien (DRKS)<br>German Clinical Trials Register<br>https://www.drks.de/drks_web/navigate.do?navigationId=search&reset=true
	2b	DRKS00011545
Protocol version (page 13 oft the manuscript)	3	Version 3.1
Funding (page 13 oft the manuscript)	4	Deutsche Gesetzliche Unfallversicherung e. V.,<br>Glinkastraße 40,<br>10117 Berlin<br>Germany
Roles and responsibilities	5a	Prof. Dr. med. Leila Harhaus<br>Senior Consultant and senior trial leader<br>Department of Hand, Plastic and Reconstructive Surgery<br>- Burn Center -<br>BG Trauma Center Ludwigshafen<br>Plastic and Hand Surgery<br>University of Heidelberg<br>BG - Unfallklinik Ludwigshafen<br>Ludwig-Guttmann-Str. 13<br>67071 Ludwigshafen<br>Germany<br><br>Dr. Martin Aman, PhD

Department of Hand, Plastic and Reconstructive Surgery
- Burn Center -
BG Trauma Center Ludwigshafen
Plastic and Hand Surgery
University of Heidelberg
BG - Unfallklinik Ludwigshafen
Ludwig-Guttmann-Str. 13
67071 Ludwigshafen
Germany

Dr. Annette Stolle
Department of Hand, Plastic and Reconstructive Surgery
- Burn Center -
BG Trauma Center Ludwigshafen
Plastic and Hand Surgery
University of Heidelberg
BG - Unfallklinik Ludwigshafen
Ludwig-Guttmann-Str. 13
67071 Ludwigshafen
Germany

Dr. Konstantin Bergmeister, PhD
Department of Hand, Plastic and Reconstructive Surgery
- Burn Center -
BG Trauma Center Ludwigshafen
Plastic and Hand Surgery
University of Heidelberg
BG - Unfallklinik Ludwigshafen
Ludwig-Guttmann-Str. 13
67071 Ludwigshafen
Germany

Dr. Arne Boecker
Department of Hand, Plastic and Reconstructive Surgery
- Burn Center -
BG Trauma Center Ludwigshafen
Plastic and Hand Surgery
University of Heidelberg
BG - Unfallklinik Ludwigshafen
Ludwig-Guttmann-Str. 13
67071 Ludwigshafen
Germany

PD Dr. Daniel Schwarz
Department of Neuroradiology,
Neurological University Clinic, Heidelberg University Hospital,
Im Neuenheimer Feld 400,

69120 Heidelberg, Germany.

Prof. Dr. Martin Bendszus
Department of Neuroradiology,
Neurological University Clinic, Heidelberg University Hospital,
Im Neuenheimer Feld 400,
69120 Heidelberg, Germany.

Prof. Dr. med. Ulrich Kneser
Head of the Department
Department of Hand, Plastic and Reconstructive Surgery
- Burn Center -
BG Trauma Center Ludwigshafen
Plastic and Hand Surgery
University of Heidelberg
BG - Unfallklinik Ludwigshafen
Ludwig-Guttmann-Str. 13
67071 Ludwigshafen
Germany

The BG Klinik Ludwigshafen is one of the largest trauma facilities and center for reconstructive and peripheral nerve surgery in Germany. MA, KB, AS and AB are part of the team. UK is a professor of Plastic Surgery and Hand Surgery at Heidelberg University and head of the department. LH is the managing senior physician of the department and has years of experience in clinical and basic research in plastic surgery. The Department of Neuroradiology of the Neurological University Clinic in Heidelberg is one of the largest centers for advanced peripheral nerve imaging in Germany. DS and MB are the head and consultant in the department leading the nerve imaging group.

(page 14 of the manuscript)

5b Deutsche Gesetzliche Unfallversicherung e. V.,
Glinkastraße 40,
10117 Berlin
Germany
Projectnumber: FR 287

5c The study sponsor is not involved in collection, management, analysis, and interpretation of data; nor in writing of the report or in the decision to submit the report for publication. They will be annually informed about the progress of the study.

	5d	The coordinating center organizes and supervises the process of data collection and data evaluation. Compliance with the schedule and patient enrollment is monitored monthly by the coordinating center. The study sponsor is informed annually. The participating institutions meet to report and accompany the study twice a year.
**Introduction**		
Background and rationale (page 3 of the manuscript: Background)	6a	An early and precise diagnosis is essential for the choice of the right therapy strategy and thus decisive for the success of the treatment in peripheral nerve injuries. Improved diagnostics using MR neurography could help to make therapy decisions more precise and thus reduce the duration of sick leave (average 26.8 days) and high therapy costs of more than 300,000 affected Europeans per year. However, there is currently no diagnostic device that can reliably correlate the anatomic-pathological parameters (e.g. partial or full lesion of the nerve) with the functional-pathological changes (muscle denervation, neuroma formation) initially and during the course of therapy. MR neurography describes a further development of conventional MR examinations which, due to the higher resolution of modern devices and innovative analysis methods, can display anatomical as well as functional parameters of nerves, providing a potential diagnostic tool of high value for the future.
(page 4 of the manuscript: Aim of the Study)	6b	We aim to determine specificity and sensitivity of MR neurography in correlation to the previous standard examinations (gold standard: clinical and intraoperative findings, neurography, and possibly sonography) of acute-traumatic nerve injuries. Furthermore, feasibility of visualization of peripheral nerves in close proximity to metal is evaluated in the osteosynthesis pilot group.<br>The patient data collection for this takes place at the respective study center (center A and center B) and the analysis of specificity and sensitivity as well as the diagnostic algorithm at center A. MR neurography is performed at center C.
Objectives (page 7 of the manuscript: Aim of the Study)	7	This prospective clinical study examines the diagnostic specificity and sensitivity of MR neurography in correlation to the previous standard examinations (gold standard: clinical and intraoperative findings, neurography, and possibly sonography) of acute-traumatic nerve injuries.<br>Furthermore, feasibility of visualization of peripheral nerves in close proximity to metal is evaluated in the osteosynthesis pilot group.

Trial design (page 4 of the manuscript: Design)	8	This study uses a prospective longitudinal design including patients at two centers in Germany. Due to ethical reasons, no randomization is performed.

Methods: Participants, interventions, and outcomes

study setting (page 4 of the manuscript: Participants)	9	Patients at two trauma centers in Germany are included. (List of study sites can be requested from the investigators.)

Eligibility criteria (page 5 of the manuscript: Background and Page 5: Participants) 10

The participants are admitted to the two burn rehabilitation centers in Germany dependent on proximity of residence and availability of a contemporary treatment capacity.

Patients with the following nerve pathologies are included:

Nerve pathologies:

- Fresh (<72h) open-traumatic nerve injuries to the trunk nerves of the upper extremity: radial, ulnar, median, musculocutaneous nerves and their branches from the brachial plexus to the distal end of the carpal tunnel.

Osteosynthesis:

- Patients with a MRT-compatible plate fixation using a titanium plate of the upper extremity.
- Humeral fracture

General inclusion criteria:

- male or female older than 18 and younger than 65 years
- communication in German or English possible
- Signed declaration of consent

Exclusion criteria:

- ejection of study participation
- Age <18 or >65 years
- Failure to show up for a follow-up examination
- Patients who are unable to provide information or who are unconscious
- Simultaneous participation in another study to evaluate a drug or medical device
- Vitally threatening injury upon initial diagnosis (e.g. multiple trauma)
- Insufficient knowledge of German or English
- mental health issues, which limits patients capacity to consent (e.g. acute psychosis, dementia)
- Pregnancy, breastfeeding
- Ongoing immunosuppressive or antineoplastic therapy

Absolute contraindications to MRI

- Pacemaker
- mechanical heart valves
- Brain and spinal cord stimulators as well as most other electrical stimulating devices implanted in the body
- Insulin pumps or other drug pumps
- ventriculoperitoneal shunts (VP shunts)
- Cochlear implants
- Foreign metal bodies in the soft tissues of the body, e.g. in the eyes, in the abdominal or chest cavity
- Obesity, which prevents the use of the MRI
- Upper limb immobility that prevents an MRI scan

In the case of a relative contraindication, only after the patient has been informed and the radiologist performing the procedure has given his consent

- Claustrophobia
- prosthetic joint replacement
- Tattoos
- Piercings
- Vascular stents, e.g. in the coronary arteries

Interventions (page 6 of the manuscript: Interventions)	11a	No intervention besides the standard procedure for nerve lesions and humerus fractures is planned.<br>The acute-traumatic nerve lesions are usually open injuries that are treated directly using epineural sutures.<br>Humerus fractures are usually treated by osteosynthesis.
(page 10 of the manuscript: Sample size)	11b	Criteria for discontinuing are met if the patient drops out voluntarily or does not show up to follow up examinations.
(page 5 of the manuscript: Participants)	11c	Patients are reminded per postal mail before each follow up appointment. Further appointments for the study are aligned to clinical follow up care.
(page 6 of the manuscript: Interventions)	11d	Participation in other studies is not allowed. No restriction of relevant concomitant care and interventions are made due to the study.

Outcomes (page 7-8 of the manuscript: Outcome measures)	12	Primary outcome of the study is the comparison of MRN and intraoperative findings in patients with nerve lesions.<br>Secondary outcome of the study are the MRN findings in patients with osteosynthesis.

Participant timeline (page 10 of the manuscript) 13

	**T1** Within 96h. after injury	**T2** 4 month after injury	**T3** 12 month after injury	**T4** 18 month after injury	[illegible]
CRF anamnesis	X	X	X	X	
Sensory and motor function testing	only healthy site	X	X	X	
**Technical examination**					
Electrophysiology		X	X	X	
MR neurography	X	X	X	X	
Neurosonography	X	X	X	X	
**Questionnaires**					
SF-36	X	X	X	X	
IES-R	X	X	X	X	
DASH	X	X	X	X	
PainDetect	X	X	X	X	
DASS	X	X	X	X	

Patients with attached osteosynthesis material

MR neurography is carried out once with special interference-suppressing analyzes, 2-12 weeks after implantation. Since these patients do not suffer from nerve lesions, no additional clinical examination or questionnaires are applied.

Sample size (page 11 of the manuscript: Sample size)	14	A sample size of N=60 patients with nerve lesions is needed. Assuming sensitivity of MR neurography to be at least 85% based on internal examinations, we therefore calculate the sample size with a 95% confidence interval using the method described by Eng J (2003):<br>$(4x\ (1.96)^{2} * 0.85 * 0.15) / (0.2^{2}) = 48.98$ patients<br>In order not to risk a loss of power with an expected dropout of approx. 10%, we add a safety margin of 10%.<br>For the group of patients with osteosynthesis no sample size calculation could be performed, since there are no data for the visualization of nerves in proximity to metal up till now.
Recruitment	15	All traumatic nerve lesions are recruited at individual centers.<br>All patients meeting inclusion criteria for patients with internal fixation of the humerus are recruited at Center A.

Methods: Assignment of interventions (for controlled trials)

Allocation:

Sequence generation	16a	Not applicable. No randomization of the intervention is planned.
Allocation conceal-ment mechanism	16b	Not applicable. No randomization of intervention is planned.
Implementa tion	16c	Not applicable. No randomization of intervention is planned.

Blinding (masking)	17a	Not applicable. No randomization of intervention is planned.
	17b	Not applicable. No randomization of intervention is planned.

Methods: Data collection, management, and analysis

Data collection methods<br><br>(page 9 of the manuscript)	18a	Data will be collected on prepared case report form (CRF) and by questionnaires and will be entered contemporary into an excel database. The trial coordinator checks regularly on the database to promote data quality (plausibility – range of values; number of missing values, duplicates etc.).<br><br>**Description of study instruments:**<br>**<u>Sensory-motor functioning</u>**<br>- Strength grades according to the classification of the British Medical Research Council from 0 = paralysis to 5 = normal strength.<br>- Hand strength using a Jamar dynamometer; Grip force and the 3-point grip are evaluated<br>- Preliminary sensory testing of both forearms and hands in a side-by-side comparison<br>- Tactile detection threshold using the Weinstein Enhanced Sensory Test (WEST), the tactile detection threshold is ascertained at index areas for each trunk nerve at the hand. Hereby, the monofilaments (200g, 4g, 2g, 0.2g, 0.07g) are placed on the skin in descending order and a defined pressure is applied.<br>- Two-point discrimination is recorded using a standardized Dellon discriminator in descending order. The distance between the pins varies from 1mm to 8mm. The static threshold is tested by placing the pins vertically on the skin and the dynamic threshold by pulling the pins over the skin.<br>- Localization of the Hoffmann-Tinel sign by lightly tapping.<br>- Range of Motion using a goniometer<br><br>**<u>Technical Examinations</u>**<br>**Electrophysiology:**<br>- <u>Motor neurography</u> comprises muscle sum action potential, distal motor latency, nerve conduct velocity.<br>- Electrode positions depending on intended nerve for measurement.<br>- Distance for DML constant 7cm

- Stimulation in steps of 5mA until supramaximal stimulus intensity is reached, then increase of 20%

- Sensory neurography comprises sensitive nerve action potential, nerve conduct velocity.
    - Electrode positions depending on intended nerve for measurement
    - Distance between different will be documented
    - Stimulation intensity will be increased in steps of 2mA until no further increase in SNAP is measured
- Somatosensory evoked potentials will be assessed using needle electrodes positioned mid-scalp
    - Positioning of stimulus electrodes will differ depending on the intended nerve for the measurement
    - Intensity of stimulation will be increased in 0,5mA steps until contractions of the muscle is seen
- Electromyography comprises quantification of pathological spontaneous activity and arbitrary activity
    - Amplitude, duration and number of phases will be described

Neurosonography:

- Neurosonography is performed using a ultrasonic probe (Siemens 14L5)

MR Neurography:

- MRT and MR Neurography measurements are performed using 3Tesla scanners (Prisma/Skyra, Fa. Siemens Healthineers)

Data management<br><br>(not mentioned in the manuscript)	19	Data entry and quality:<br>For data entry a digital form was created with Microsoft Excel. For each variable, input characteristics are defined such as string or numeric, in order to minimize failures during data entry. On a regularly basis data will be checked with SPSS about double entries, displaying maximum and minimum values and frequency of missing values. Date entry will be performed contemporary by a research assistant therefore errors during completing of the CRF will be noticed soon. In case of an error the research assistant will confer with the clinician who filled in the form (4-eye-principle).<br><br>Data security:<br>CRF's, questionnaires and the data base contain a numeric code instead of patient names. CRF's and questionnaires are kept in a locked filing cabinet to which only the project leader, study coordinator and the research assistant have access.<br>A coding list is kept for assignment at follow-up, only project manager, study coordinator and research assistant have access. The coding list is kept apart from CRF's, questionnaires and the data base.<br><br>All information can also be found in the study protocol.

Statistical methods (page 11 of the manuscript: Statistical methods)	20a	MR neurography diagnosis of nerves will be represented as a numerical value between 0 and 1 using fractional anisotropy. The Pearson correlation coefficient is used to correlate the FA with the intraoperative findings (% of the severance). We consider values between 0.7 and 1 a strong correlation, values from 0.5 to 0.7 a moderate one and from 0.3 to 0.5 a weak correlation.<br>A T-test is used to determine the dependence of the FA on the clinical and neurosonographic binary variables (presence of sensory deficit, presence of motor deficit, presence of a defect in neurosonography), provided the data are normally distributed. Otherwise, corresponding non parametric tests are used. In order to examine variables over time an ANOVA for dependence of FA change on the clinical-binary parameters will be performed (resp. corresponding non parametric test). Relation between changes of FA values with changes in nerve conduction velocity will be tested using partial correlation coefficient. Due to the hypothesis-generating study design of this pilot study, no correction for multiple testing is applied [15]. A p-value of 0.05 or less is established as statistical significance in accordance with Good Scientific Practice for all investigations. The study results are evaluated with SPSS V21 software from IBM.
(page 11 of the manuscript: Statistical methods)	20b	Results of DASH, PainDetect, IES-R, SF-36 and DASS will be expressed by means and standard deviation.

(page 11 of the manuscript: Statistical methods)	20c	Only datasets with a minimum of one follow up assessment will be included in the analysis.<br><br>A drop-out rate of 20% usually expected in a clinical trial is calculated in the sample size. But we expect a lower drop-out rate of 10%, as patients are usually closely connected to peripheral nerve centers. Drop-out patients as well as non-participants will be assessed with age, sex and type of injury checked for systematic differences with the study population.<br><br>In questionnaires, we will allow a maximum of 10 % missing data, which will be replaced by imputation, but only if 50% missing data are not exceeded per scale. No missing data are expected in the CRFs, as the completeness is checked immediately after data entry.
**Methods: Monitoring**		
Data monitoring<br><br>(not mentioned in the manuscript)	21a	The data monitoring committee (DMC) is composed of members of the participating institutions and members of the sponsor and. The DMC checks annually the progress and compliance with the timeframe. A written report is submitted to the sponsor every year. Beside of control of the time frame the sponsor is not involved in data collection or monitoring.
(interim analysis and stopping rules)	21b	Termination of the study is planned, if severe side effects of the assessments occur (e.g. side effects of contrast agent), responsibility for this decision rests with the senior and junior leaders of the study (sponsor is to be informed).<br>Interim analysis of the data will be performed annually and reported to the sponsor and the members of the research group.

Harms<br><br>(page 7 of the manuscript: Interventions	22	Only standard treatment is planned, no new interventions will be applied. Solicited and spontaneously reported adverse events or unintended effects of the assessments such as claustrophobia or allergic reactions to the contrast agent will be recorded. These cases will also be documented in the CRF's of the patients and reported to the sponsor, the study team and the data monitoring team. A report of adverse events will be also part of publication.<br>Should there be any adverse effects due to the assessment for the study the patient will be administered immediately to the doctor of duty.
Auditing<br><br>(not mentioned in the manuscript)	23	No external auditing of the trial is planned.<br>CRF's will be proofed regularly for completeness and plausibility during data entry.

Ethics and dissemination

Research ethics approval<br><br>(page 14 of the manuscript: Ethical approval)	24	The study is approved by the federal ethics committee of Rhineland-Palatinate. Participants must give written consent prior of inclusion to the study.
Protocol amendments<br><br>(not mentioned in the manuscript)	25	In case of trial modification this will be reported to the ethics committee, from which approval is obtained formally. Further, the clinical trial register will be informed about changes, as well as the sponsor and the investigation team.

Consent or assent<br><br>(page 4 of the manuscript)	26a	Potential participants of the study will be given detailed information about the trial in written form as well as orally by one of the research members. If they are willing to participate, informed consent will be obtained by the senior or junior study leader. The consent will be signed by the participant and by the study leader. The participant will receive a copy. The original will be archived by the senior study leader. No under aged persons or not competent persons will be included in the trial.
	26b	Data collected will not be used in ancillary studies or promoted to another party.
Confidentiality<br><br>(not mentioned in the manuscript)	27	Information for the study will be collected on CRF's and questionnaires which contain a numeric code. Due to assignment of the data of the different assessment points during the study a code list will be maintained. This list will be kept strictly separate to the CRF's and the database in a locked cabinet, only the project leader, study coordinator and the research assistant have access.<br>Informed consents of the participants will be stored apart from other study documents in a locked file cabinet at each study center, only the project leader of the center will have access.<br>For joint analysis data of center B and C must be transferred to the coordinating center. The anonymized data set will be transferred by encrypted file on a stick (protected by password) by registered mail.<br><br>After finishing the enrolment and study assessment study data will be stored 10 years (along data privacy act of Germany), after that period the files will be irretrievably destroyed.
Declaration of interests<br><br>(page 14 of the manuscript:	28	There are no financial or competing interests of the investigators for the study.

Competing interests)		
Access to data<br><br>(not mentioned in the manuscript)	29	The investigators involved have access to the anonymized dataset. The sponsor will be allowed to view the anonymized dataset and the protocol of the analysis. The data will be deleted after a retention period of 10 years.
Ancillary and post-trial care<br><br>(not mentioned in the manuscript)	30	Participant insurance has been set up with 100.000€ per participant.
Dissemination policy<br><br>(not mentioned in the manuscript)	31a	The trial results will be published in corresponding journals and conferences to healthcare professionals, the public. The sponsor will receive a final report.
	31b	At publication of the study only authors are named, which will have substantial contributions to the study design, patients' enrolment and assessment, data management and analysis.<br>No use of professional writers is planned.
	31c	The full study protocol, the CRF's and the statistical code can be requested by interested fellow researchers from the senior study leader. No public access to the participant-level dataset is planned.

Appendices

Informed consent materials	32	Information about the study<br>Consent form
Biological specimens	33	Not applicable. There is no collection of biological specimens.
